# Prevalence of *Leucocytozoon* infection in domestic birds in Ghana

**DOI:** 10.1371/journal.pone.0294066

**Published:** 2023-11-29

**Authors:** Constance Agbemelo-Tsomafo, Samuel Adjei, Kwadwo A. Kusi, Kirk W. Deitsch, Daniel Amoah, Richard Obeng-Kyeremeh, Ayishetu M. Sumabe, Yaw Aniweh

**Affiliations:** 1 Department of Biochemistry, Cell, and Molecular Biology, College of Basic and Applied Sciences, University of Ghana, Accra, Ghana; 2 West African Centre for Cell Biology of Infectious Pathogens, College of Basic and Applied Sciences, University of Ghana, Accra, Ghana; 3 Department of Animal Experimentation, Noguchi Memorial Institute for Medical Research, College of Health Sciences, University of Ghana, Accra, Ghana; 4 Department of Immunology, Noguchi Memorial Institute for Medical Research, College of Health Sciences, University of Ghana, Accra, Ghana; 5 Department of Microbiology and Immunology, Weill Cornell Medical College, Cornell University, New York, NY, United States of America; Temple University, UNITED STATES

## Abstract

*Leucocytozoon* is a haemosporidian parasite known to cause leucocytozoonosis in domestic and wild birds in most parts of the world. It is an important pathogen, as some species can be pathogenic, especially in domestic birds. One of the factors affecting poultry health management worldwide is parasitism. However, the study of haemosporidian parasites in Ghana is still lacking. This study sought to assess the prevalence and diversity of *Leucocytozoon* parasites in domestic birds in Ghana. Blood samples were collected from domestic birds in Ghana’s Bono and Eastern regions to screen for *Leucocytozoon* parasites. Thin blood smears were prepared for microscopy and DNA was extracted from whole blood kept in ethylenediaminetetraacetic acid (EDTA) tubes for PCR. Due to the large number of samples, real-time PCR was performed to amplify the conserved rDNA gene. Two different nested PCR protocols were performed on the positive samples obtained from real-time PCR results, to amplify a partial region of the mitochondrial cytochrome b gene and the amplicons were sequenced. Sequencing revealed six new lineages of *Leucocytozoon* sp. recovered in 976 individual domestic birds and these sequences were deposited in the National Center for Biotechnology Information (NCBI) GenBank. An overall *Leucocytozoon* prevalence of 11.6% was reported in all birds sampled. The most prevalent lineage LGHA146 (GenBank accession no. OM643346) (93.8%) was found infecting 3 bird species, *Gallus gallus*, *Meleagris gallopavo*, and *Anas platyrhynchos*. Phylogenetic analysis revealed that the new lineages (GenBank accession nos. OM643342, OM643343, OM643344, OM643345, OM643346, and OM643347), reported in this study were closely related to *Leucocytozoon schoutedeni*. We suggest that further studies be conducted to evaluate the effect of these parasite species on the general well-being of poultry in Ghana.

## Introduction

*Leucocytozoon* parasites are protozoans belonging to the phylum Apicomplexa [1] and order Haemosporida. The genus belongs to the avian haemosporidian parasites group, which also includes the genera, *Haemoproteus*, *Plasmodium*, and *Fallisia* [2]. Similar to other haemosporidian parasites, *Leucocytozoon* species exhibit a complex life cycle using two hosts with merogony occurring in vertebrate hosts, and sporogony in the vector; simuliid (Simuliidae) flies or culicoides midges [2]. These parasites infect both wild and domestic birds in many parts of the world. Symptoms include listlessness, green faeces, pale comb, anorexia, and anaemia, resulting in increased mortality and decreased egg production. Outbreaks of leucocytozoonosis disease in poultry have been reported in several countries including Taiwan [3, 4], Japan, Philippines, Singapore, Malaysia and Thailand [4], Myanmar [5], and Korea [6, 7]. In Africa, there have also been reports of *L*. *schoutedeni* in chickens in South Africa [8], Uganda, and Cameroon [9], and other species of *Leucocytozoon* in wild birds from West Africa [10, 11].

*Leucocytozoon* usually presents subclinical infections, however, certain species could cause clinical symptoms leading to fatal disease [12]. *Leucocytozoon caulleryi* reportedly caused severe damage to the poultry industry in Japan due to anemia, hemorrhage, mortality in young chickens, reduced egg production, and laying of soft-shelled eggs in layer hens [13]. Poultry health is of great importance in securing and sustaining food security. In Ghana, poultry production impacts the country’s economy greatly, accounting for up to 14% total gross domestic product [14]. The major source of protein for Ghanaians is chicken [14], and other poultry products. It is therefore paramount to ensure quality poultry is being produced for the benefit of the consumers. Apart from viral and bacterial diseases of poultry that have been studied quite extensively in Ghana, there are very few documented reports on blood parasites. In monitoring the parasites that can affect poultry health, it has been established that parasites such as *Leucocytozoon* are of great importance. So far, about 45 morphologically distinct species of *Leucocytozoon* parasites [15] have been described for causing leucocytozoonosis disease in wild and domestic birds. Only a few of these species have, however, been described in poultry. Previous studies described *Leucocytozoon simondi*, and *L*. *smithi*, in waterfowls and turkeys respectively, *L*. *caulleryi*, *L*. *sabrazesi* (*L*. *macleani*), and *L*. *schoutedeni* in domestic chickens [5–7]. Infections in domestic chickens could be as high as 56% [1]. Despite the reports of *Leucocytozoon* infections in wild and domestic birds around the globe, there is little information about these infections in Ghana. Knowledge of the epidemiology of these parasites in the Ghanaian poultry industry will help in planning strategies for disease surveillance and monitoring. This study focused on *Leucocytozoon* prevalence in the Ghanaian poultry industry and how they are genetically related to other *Leucocytozoon* species around the globe. Our study confirmed *Leucocytozoon* infections in poultry in Ghana and successfully showed that these parasites are closely related to *L*. *schoutedeni*.

## Materials and methods

### Ethical approval

This study was carried out using protocols approved by the Institutional Animal Care and Use Committee (IACUC) of the University of Ghana (protocol code UG-IACUC 003/20-21).

### Sample collection and smear preparation

Sample collection took place between November and December 2019 and 2020 in five poultry communities, Dumasua, Kantro, Fiapre, and Chiraa in the Bono region, and Aburi in the Eastern region of Ghana.

About two milliliters (~2 ml) of venous blood was drawn from the brachial veins of individual birds. The blood was drawn using a 2ml syringe with a 21G needle for small birds and a 23G needle for bigger birds and dispensed into 2ml EDTA tubes and mixed carefully to thoroughly mix blood with anticoagulant. Before blood collection, the brachial site was aseptically disinfected using a cotton ball soaked with 70% ethanol. A fresh syringe and needle were used for each bird to prevent cross-contamination of samples. After drawing blood, a dry cotton ball was used to clean the bleeding site and gentle pressure was applied to completely stop bleeding. The sampled birds were marked to prevent recapture. 2–3μl of non-anticoagulant blood was deposited from the syringe directly onto a clean grease-free appropriately labeled microscope slide and smeared thinly to make a thin blood smear. The smear was air-dried and immediately fixed in absolute methanol for 1-3min and air-dried in the field. Three thin blood smears were prepared for each bird. The fixed smears were transported to the laboratory and stained with 10% Giemsa solution for 20min, rinsed with tap water, and air dried. The Blood in EDTA tubes was kept on ice without direct contact with the ice and transported to the laboratory for further processing. The blood samples were kept at -20°C in the laboratory before other analyses.

### Traditional microscopy

The stained slides were examined at 1000X magnification using the 100X oil immersion objective lens of an Olympus CH30 microscope. At least 100 microscopic fields were screened for *Leucocytozoon* parasites.

### Molecular analyses

Deoxyribonucleic acid (DNA) was extracted from whole blood using a DNeasy extraction kit (Qiagen, Valencia, CA, USA) according to the manufacturer’s protocol. The presence of DNA was confirmed by running 2 μl of the extract on 1.5% agarose gel electrophoresis, post-stained with Diamond Nucleic Acid dye (Promega Ribose Nucleic Acid corporation) and visualized under ultraviolet light (UV).

### Real-time PCR

All samples were screened first using a real-time PCR protocol that amplified 182 bp of the ribosomal DNA (rDNA) conserved in all three haemosporidian genera (*Leucocytozoon*, *Haemoproteus* and *Plasmodium*) using the primers R330F (5’- CGTTCTTAACCCAGCTCACG - 3’) and R480RL (5’- GCCTGGAGGTWAYGTCC - 3’) [16]. All reactions were carried out using a 2x Luna Universal qPCR master mix in a real-time thermocycler (ABI 7300). The total volume of the reactions was 10 μl, with 5 μl of 2x Luna qPCR master mix, 0.4 μl of each primer (10 μM concentration), 2.2 μl of molecular grade water, and 2 μl of DNA template. The following cycling conditions were used to run the reaction: 95°C for 3min, followed by 40 cycles of 95°C for 15s and 53°C for 35s (with a plate read) followed by a dissociation analysis using instrument default settings. Positive and negative controls were included in all runs. The positive controls were samples that tested positive from previous studies and sterile nuclease-free water was used as a negative control. For verification of procedure, real-time PCR products were run on 1.5% agarose gel, post-stained with Diamond Nucleic Acid dye (Promega corporation), and visualized under ultraviolet light (UV). The band sizes of 182bp [17] confirmed positive samples.

### Nested PCR

To amplify the mitochondrial cytochrome b gene, all samples that tested positive for real-time were screened using a nested PCR that amplifies 480bp of the cyt b gene. The primers HaemNFI (5’-CATATATTAAGAGAAITATGGAG-3’) and HaemNR3 (5’-ATAGAAAGATAAGAA ATACCATTC- 3’) were used in the first round of nested PCR and HaemFL and HaemR2L for the second round [18]. All reactions were carried out using One Taq quick load 2x master mix on a thermocycler (ABI 2720). The total volume of the reactions was 10 μl, with 5 μl of the master mix, 0.6 μl of each primer (10 μM concentration), 1.8 μl of molecular grade water, and 2μl of DNA template. The following cycling conditions were used to run the reaction: 95°C for 3 minutes, followed by 20 cycles of 95°C for 30sec, 50°C for 30sec, 68°C for 1min, and a final elongation at 68°C for 5 minutes and holding at 4°C. For the second round of PCR, 2 μl of amplicon from the first round was used and the number of cycles was increased to 35. Positive controls were not included because all the samples tested were positive from real-time PCR. Sterile nuclease-free water was used as a negative control in place of the DNA template. The amplified DNA (3μl) was then submitted to electrophoresis on a 1.5% agarose gel and detected by post-staining with diamond nucleic acid dye (Promega corporation), and UV trans-illumination. The expected target size was 480 bp and the band size was measured using a 50bp DNA ladder (Biolabs).

### Nested PCR with modification

The first nested PCR protocol used failed to amplify many known positive samples even after repeating the procedure three times. The nested PCR with modification was used to amplify a shorter region of the mitochondrial cyt b gene. The first round of the nested PCR with modification was performed using the same primers and conditions used in the previous nested PCR run. However, the second round was performed using the primers L545F (5’- ACAAATGAGTTTCTGGGGA-3’) and L825R (5’–GCAATTCCAAATAAACTTTGAA–3’) [16] to amplify a partial region of the mitochondrial cytochrome b gene of the *Leucocytozoon* genus, using the same cycling conditions used previously in the second round of nested PCR.

The PCR products were analyzed by gel electrophoresis following the previous protocol. The expected target size of 280 bp was measured using a 50 bp DNA ladder (Biolabs).

### Sequencing and phylogenetic analysis

The positive amplicons were shipped to Macrogen sequencing company (Macrogen, Europe) for bidirectional Sanger sequencing using the primers L545F and L825R. The resulting sequences were edited in Chromas (Version 2.6.6) and assembled in Bioedit (Version 7.2.5). Consensus sequences generated were submitted against the MalAvi [19] and National Center for Biotechnology Information (NCBI) databases for comparison to existing sequences. The new *Leucocytozoon* lineages identified in this study were deposited into the NCBI database and assigned Genbank accession numbers (Table 2). Closely related sequences were retrieved from the NCBI GenBank and Malavi database, added to the sequences found in this study, and subjected to multiple alignments using the Multiple Alignment using Fast Fourier Transform (MAFFT) [20] online tool (MAFFT version 7). Multiple aligned sequences were trimmed in Bioedit, and phylogenetic analysis was performed using Molecular Evolutionary Genetics Analysis 11 (MEGA 11) software [21]. The best model analysis (TN93+G) was found, and a maximum likelihood tree was constructed in MEGA 11 with 1000 bootstrap replications, and the tree was edited using interactive Tree of Life (iTOL) [22].

### Statistical analyses

All statistical analyses were performed using GraphPad PRISM (version 5.01) and Microsoft Excel. The Kruskal-Wallis test was used to compare *Leucocytozoon* infections among different bird species.

## Results

A total of 976 individual domestic birds belonging to four species were sampled and screened for *Leucocytozoon* parasites (Table 1). An overall prevalence of 11. 6% based on nested PCR alone was recorded among all birds sampled and species-specific prevalence ranged from 3.3% to 17.7% (Table 1).

**Table 1:** *Leucocytozoon* parasite prevalence was recorded in various bird species.

Bird Species Common/scientific name	No. sampled	No. detected (%) (Nested PCR)	No. detected (%) (Nested PCR with modification)
Domestic fowl/*Gallus gallus domesticus*	618	1 (0.1)	71 (11.5)
Domestic turkey/ *Meleagris gallopavo domesticus*	90	-	16 (17.7)
Domestic mallard/*Anas platyrhynchos domesticus*	178	-	24 (13.5)
Common quail/*Coturnix coturnix*	90	-	3 (3.3)
	976	1(0.1)	114 (11.6)

%, *Leucocytozoon* prevalence No., number.

*Leucocytozoon* prevalence was higher in *M*. *gallopavo* compared to all other three bird species (Table 1). A pairwise comparison using Dunn’s test indicated that *Leucocytozoon* prevalence was significantly higher in *M*. *gallopavo* than in *C*. *coturnix* (P = 0.0195).

Microscopy screening of thin blood smears for morphological identification of *Leucocytozoon* sp. did not reveal any gametocyte stage of the parasite. Whereas nested PCR using the primers HaemFL and HaemR2L on qPCR positive samples was not very sensitive in confirming the positive samples (S1 Fig), amplification of a shorter fragment using L545F and L825R primers was more sensitive (S2 Fig).

There were six different *Leucocytozoon* lineages found infecting the birds sampled and sequences from these lineages have been deposited in the NCBI GenBank database and given accession numbers OM643342-OM643347 (Table 2). The sequences in this study were compared with six related sequences from the Malavi database and twelve sequences from the NCBI GenBank (Table 2).

**Table 2:** New *Leucocytozoon s*p. lineages from Ghana and closely related lineages recovered from NCBI GenBank and Malavi database.

SEQUENCE NAME (SOURCE)	ACCESSION No.	Country
*LGHA29Q* (this study)	OM643342	Ghana
*LGHA73* (this study)	OM643343	Ghana
*LGHA83* (this study)	OM643344	Ghana
*LGHA111* (this study)	OM643345	Ghana
*LGHA146* (this study)	OM643346	Ghana
*LGHAAS2* (this study)	OM643347	Ghana
*L*. *schoutedeni GALLUS07* (Malavi)	DQ676824	Uganda
*L*. *schoutedeni GALLUS06* (Malavi)	DQ676823	Uganda
*Leucocytozoon sp*. *isolate AS46132* (NCBI)	MK066379	Thailand
*Leucocytozoon sp*. *isolate CKSK8* (NCBI)	MT500537	Thailand
*Leucocytozoon sp*. *isolate CKKB19* (NCBI)	MT500532	Thailand
*Leucocytozoon sp*. *isolate AS46129* (NCBI)	MK066376	Thailand
*Leucocytozoon sp*. *isolate AS46146* (NCBI)	MK066377	Thailand
*Leucocytozoon sp*. *isolate CBPR21* (NCBI)	MT500534	Thailand
*Leucocytozoon sp*. *isolate CKKB24* (NCBI)	MT500533	Thailand
*Leucocytozoon sp*. *isolate CKKB10* (NCBI)	MT500531	Thailand
*L*. *schoutedeni isolate K3* (NCBI)	MW043726	Thailand
*L*. *schoutedeni isolate C06* (NCBI)	MW043725	Thailand
*L*. *schoutedeni isolate K12* (NCBI)	MW043728	Thailand
*L*. *schoutedeni isolate B09* (NCBI)	MW043724	Thailand
*GALLUS26* (Malavi)	MW043727	Thailand
*GALLUS42* (Malavi)	MN907103	Thailand
*GALLUS45* (Malavi)	MN907106	Thailand
*GALLUS23* (Malavi)	LC550049	Myanmar
*L*. *schoutedeni isolate LschMH02* (NCBI)	KT290937	Malaysia

**Fig 1:**
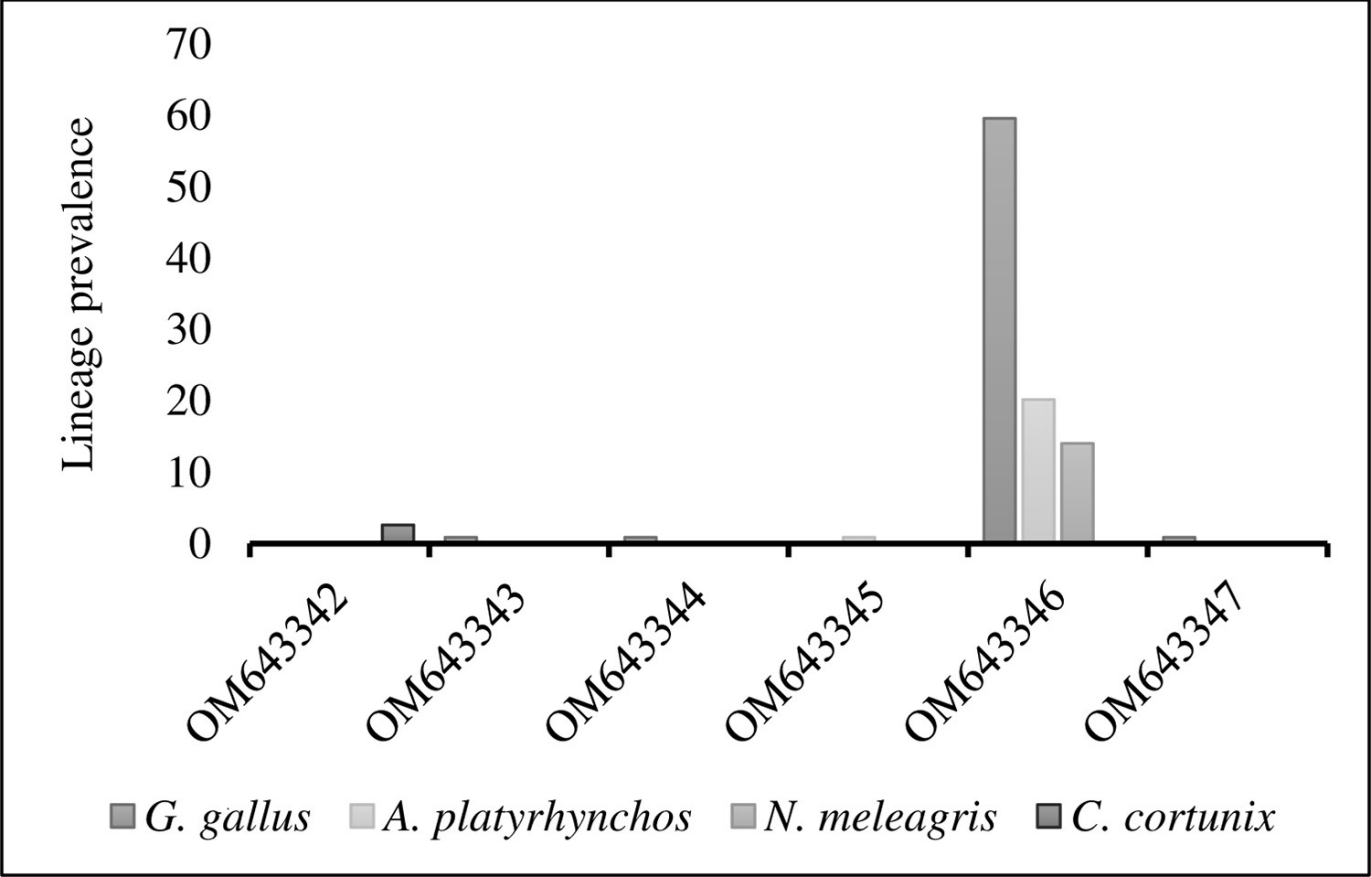
Prevalence of *Leucocytozoon* sp. lineages in different bird species. Accession numbers of lineages are shown on the x-axis and lineage prevalence is on the Y-axis.

Four *Leucocytozoon* lineages, LGHA73 (OM643343), LGHA83 (OM643344), LGHA146 (OM643346), and LGHAAS2 (OM643347) were found in domestic chickens (*G*. *gallus*), two lineages LGHA111 (OM43345) and LGHA146 (OM643346) were recorded in *A*. *platyrhynchos* and LGHA29Q (OM643342) was recorded only in *C*. *coturnix* (Fig 1). The most prevalent lineage LGHA146 (OM643346) was found in three bird species *G*. *gallus*, *A*. *platyrhynchos*, and *N*. *meleagris*. The lineage LGHA146 (OM643346), the most prevalent (93.8%) among the six lineages reported had a prevalence of 59.6%, 20.17%, and 14.03% in *G*. *gallus*, *A*. *platyrhynchos*, and *M*. *gallopavo* respectively (Fig 1). The lineage LGHA29Q (OM643342) which was found only in *C*. *coturnix* recorded a 2.6% prevalence (Fig 1).

**Fig 2:**
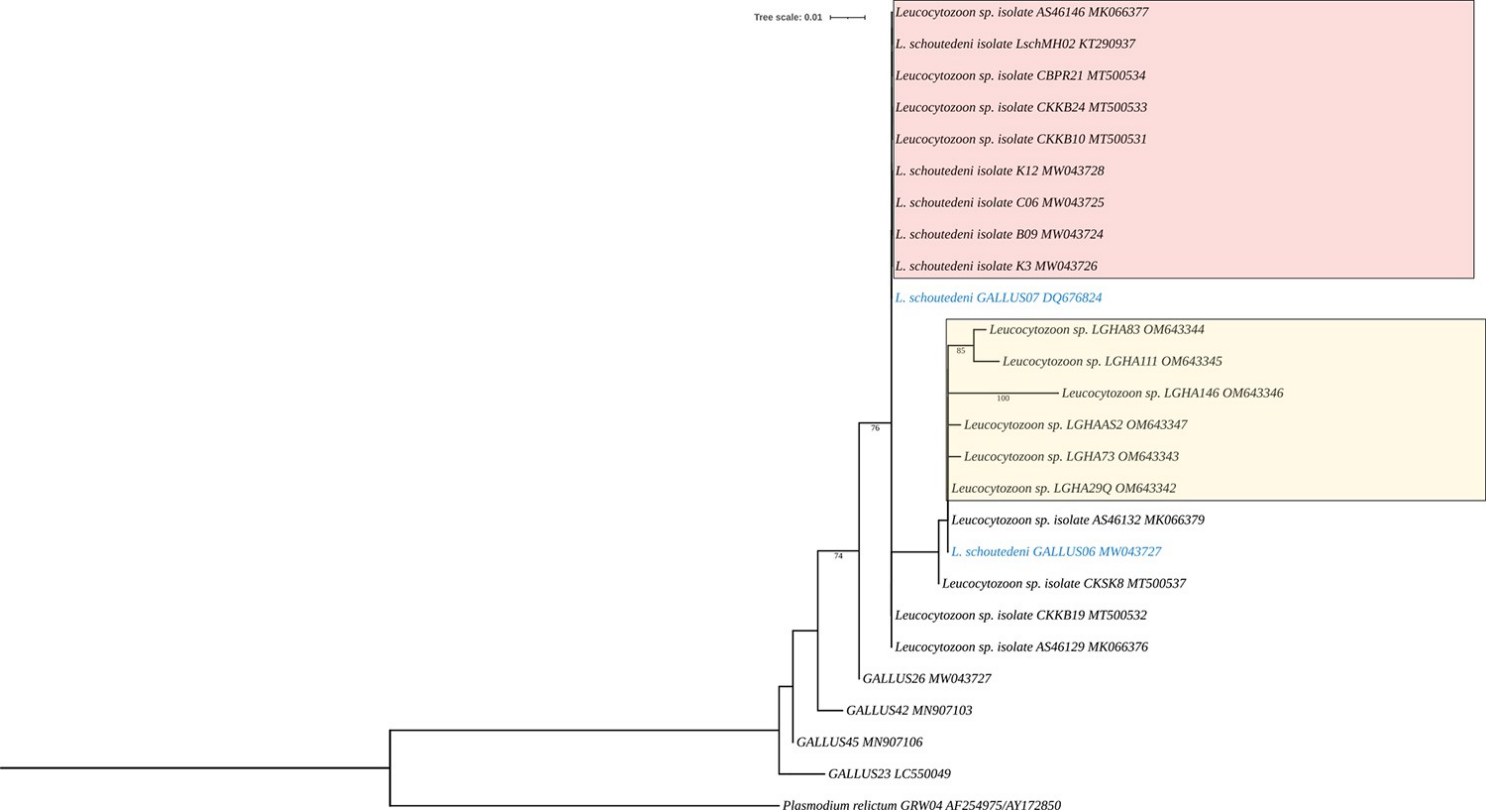
A maximum-likelihood phylogenetic tree constructed with partial mitochondrial cytochrome *b* gene sequences. Six *Leucocytozoon* lineages (shown in the yellow box) found in this study were used together with others obtained from the NCBI GenBank and Malavi databases. Lineage names in the pink box and black text without the box represent related *Leucocytozoon* sequences from South Asia. Lineage names in blue text represent *L*. *schoutedeni* found in Uganda. Values on nodes denote bootstrap analysis based on 1000 replicates. *Plasmodium relictum GRW04* (AF254975/AY172850) was used to root the tree.

*Leucocytozoon* lineages from this study cluster together and form a monophyletic group with *L*. *schoutedeni* GALLUS06 from Uganda in East Africa, and *Leucocytozoon sp*. *isolate AS46132 and Leucocytozoon sp*. *isolate CKSK8* both from Thailand in South Asia. These groups show a more recent common ancestor than the other groups suggesting fewer evolutionary changes compared to the other groups. Monophyly was also observed between LGHA83 and LGHA111 from Ghana (this study) suggesting that they share some unique characters. All the *Leucocytozoon* species lineages found in Ghana have clustered together and form a monophyletic group with *L*. *schoutedeni* lineages from East Africa and South Asia (Fig 2).

## Discussion

Research on *Leucocytozoon* has become very important in all parts of the world due to its economic importance in poultry farming. This study is the first report of *Leucocytozoon* prevalence in poultry in Ghana. Six partial cytochrome b lineages of *L*. *schoutedeni* have been identified in poultry in Ghana for the first time and deposited in the NCBI GenBank.

The new genetic lineages of *L*. *schoutedeni* were found in domestic fowl, turkey, duck, and quail. Contrary to these findings, *L*. *schoutedeni* has been documented only in domestic fowl (*G*. *gallus*) in Kenya [23], Cameroon, and Uganda [9]. Our study did not find any morphological stages of the *L*. *schoutedeni* parasite lineages identified by PCR. Thin blood smears were well prepared and of good quality, therefore the undetected blood stages of the parasites cannot be attributed to the poor quality of blood smears. It is highly possible that due to the extremely low parasitemia in these naturally infected birds, we could not find any gametocytes of the parasite. Nevertheless, this observation could also be a result of the incomplete life cycle of the parasites within the host causing the detection of sporozoites DNA in the host [15] suggesting abortive infection similar to reports on *Haemoproteus minutus* [24]. This notwithstanding, it is also likely that gametocytes were simply undetected by microscopy due to difficulty in proper morphological identification of the gametocytes.

The lineages of the parasite described in this study were of shorter fragment length compared to the previously described 480bp region of the mitochondrial cyt b gene [18]. This is because of the lower sensitivity of the nested PCR that amplifies the 480bp region of the mitochondrial cyt b gene on our samples. The qPCR that amplifies the conserved rDNA gene of the haemosporidian parasites genera *Plasmodium*, *Leucocytozoon*, and *Haemoproteus* [17], was used to select positive samples. When these samples were retested using the nested PCR described by Hellgren et al. [18] to amplify the mitochondrial cytochrome b gene for better comparison with previously described sequences, only 0.1% of the samples were positive suggesting lower sensitivity of this protocol to our samples. However, when the primers suggested by Lutz et al. [16] were used, 11.6% of the samples were detected as positive, thereby contradicting the findings reported in the studies by Bell et al. [17]. These results suggest that the *Leucocytozoon* prevalence in Ghanaian poultry farms has been underestimated in this study. New primers should be designed, and thorough studies conducted to confirm the prevalence of these parasites. The reason behind these variations is not clear, however, it is recommended that the whole cytochrome b gene of these parasites should be sequenced in future studies for a thorough study.

The overall prevalence of *Leucocytozoon* parasite infection recorded in this study was 11.6%. which was lower than *L*. *schoutedeni* prevalence of 52.1% in Kenya [23], 50% in Tanzania [25], and 31% in Uganda [9] reported in domestic birds. Other *Leucocytozoon* species reported in other African countries included a 3% and 1% prevalence of *L*. *sabrazesi* in young and adult chickens respectively, in Zimbabwe [26], and 34% in Ibadan, Nigeria [27]. Earlier studies recorded no *Leucocytozoon* infection in Ghana [28, 29]. The current prevalence of 11.6% is closer to the 15% prevalence reported in village chickens in Gombe state, Nigeria [30]. The differences in infection rates in various geographical locations could be attributed to the availability of insect vectors and the extent of sampling.

The overall *Leucocytozoon* prevalence of 17.7% in turkeys (meleagrinidae) was statistically higher than the 3.3% in quail (*C*. *coturnix*). This variation could be attributed to the fact that turkeys were mostly infected by the most prevalent lineage of the parasite. This suggests the possibility of insect vectors carrying the prevailing lineage more than the other lineages.

The phylogenetic tree (Fig 2) shows a close evolutionary relationship between our sequences and *L*. *schoutedeni* from Uganda and South Asia. The six new *Leucocytozoon* parasite lineages LGHA146, LGHA29Q, LGHAAS2, LGHA73, LGHA111, and LGHA83, described in this study form a clade that is closely related to *L*. *schoutedeni*, a domestic bird parasite, previously found in Africa [9, 23] and South Asia [3, 4, 6, 7].

The results of this study suggest that the *Leucocytozoon* parasites reported in Ghana are new lineages of *L*. *schoutedeni*. Microscopy results in future studies are needed to confirm these parasites.

It is not known how the presence of *Leucocytozoon* parasites could affect poultry in Ghana. Following this first report of Leucocytozoonosis in Ghana, further studies will be useful to ascertain the economic importance of these parasites in the Ghanaian poultry industry. Further studies need to be carried out to establish the impact of *L*. *schoutedeni* infections on the Ghanaian poultry industry.

## Conclusion

In conclusion, an overall *Leucocytozoon* prevalence of 11.6% was recorded in domestic birds sampled in Ghana. Six new *Leucocytozoon* lineages were reported among the birds sampled, with LGHA146 (OM643346) being the most prevalent (93.8%) lineage occurring. The lineages found in this study were closely related to *L*. *schoutedeni*. The findings of this study have confirmed Leucocytozoonosis disease in Ghanaian poultry. It is important to further study the most prevalent lineage of the parasite using transcriptomics to assess the impact of these parasites on the birds at the molecular level and its effect on the general well-being of the birds.

## Supporting information

S1 Fig Nested PCR. Agarose gel electrophoresis on positive samples from qPCR showing amplification of 480 bp partial region of the mitochondrial Cyt b gene of avian *Leucocytozoon*. Ld = 50bp DNA ladder, wells 1–16 = positive samples from qPCR run which tested negative for *Plasmodium* and *Haemoproteus* genera.
(TIF)

S2 Fig Nested PCR with modification. Agarose gel electrophoresis on positive samples from qPCR showing amplification of 280bp fragment of the mitochondrial Cyt b gene of avian *Leucocytozoon*. Ld = 50bp DNA ladder. Wells 1–23 positive test samples from qPCR. well 24 = negative control.
(TIF)
