# Supplementary figures and images for: Prevalence of Leucocytozoon infection in domestic birds in Ghana
Source: PLoS One. 2023 Nov 29;18(11):e0294066. doi: 10.1371/journal.pone.0294066 (PMC10686479; doi:10.1371/journal.pone.0294066)

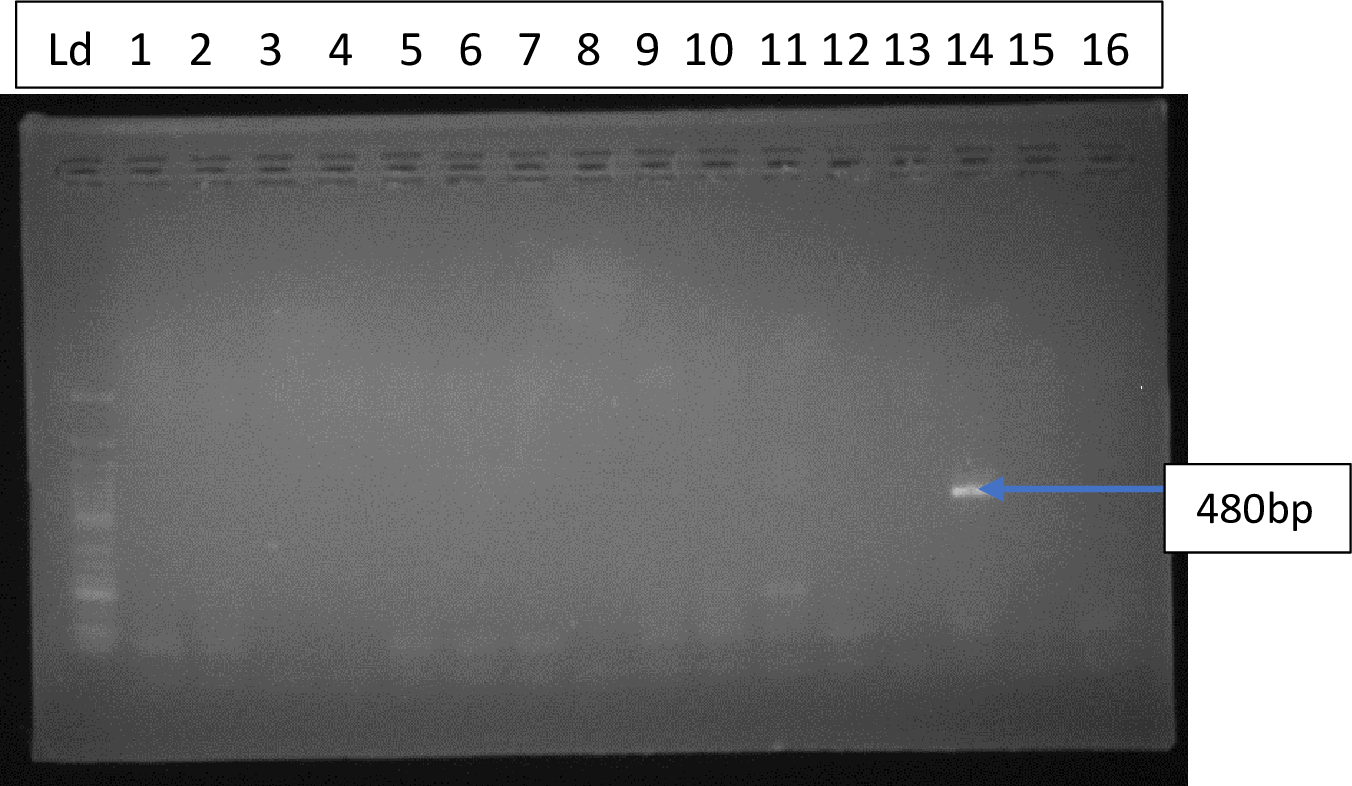

Supplement: S1 Fig — Agarose gel electrophoresis on positive samples from qPCR showing amplification of 480 bp partial region of the mitochondrial Cyt b gene of avian Leucocytozoon. Ld = 50bp DNA ladder, wells 1–16 = positive samples from qPCR run which tested negative for Plasmodium and Haemoproteus genera. (TIF) [file pone.0294066.s001.tif]

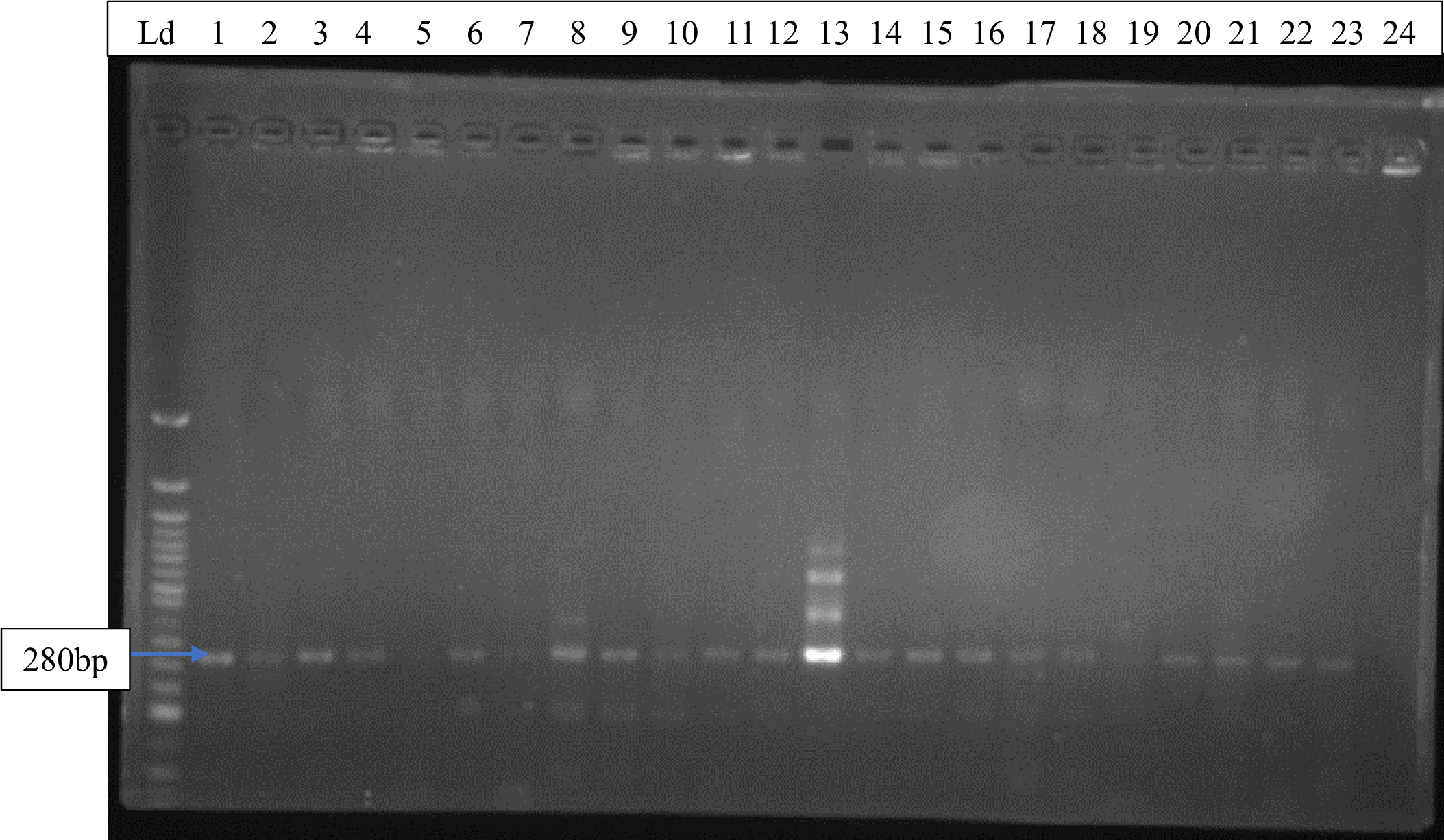

Supplement: S2 Fig — Agarose gel electrophoresis on positive samples from qPCR showing amplification of 280bp fragment of the mitochondrial Cyt b gene of avian Leucocytozoon. Ld = 50bp DNA ladder. Wells 1–23 positive test samples from qPCR. well 24 = negative control. (TIF) [file pone.0294066.s002.tif]
